# The effects of potatoes and other carbohydrate side dishes consumed with meat on food intake, glycemia and satiety response in children

**DOI:** 10.1038/nutd.2016.1

**Published:** 2016-02-15

**Authors:** R Akilen, N Deljoomanesh, S Hunschede, C E Smith, M U Arshad, R Kubant, G H Anderson

**Affiliations:** 1 Department of Nutritional Sciences, Faculty of Medicine, University of Toronto, Toronto, Ontario, Canada

## Abstract

**Background:**

The effect of carbohydrate (CHO) foods on blood glucose (BG) is ranked by their glycemic index (GI). Boiled and mashed potatoes (BMPs) are ranked as high GI foods, whereas pasta and rice have moderate GI rankings. The objective of this study was to compare *ad libitum* consumption of common CHO dishes consumed with meat on meal-time food intake and post-meal satiety, BG, insulin and gut hormones in 11- to 13-year-old normal weight children.

**Methods:**

Two randomized crossover studies were conducted. At weekly intervals, children (experiment 1: 12 males (M), 8 females (F); experiment 2: 6M, 6 F) received in random order 1 of 5 CHO side dishes of rice, pasta, BMP, fried French fries (FFF) or baked French fries (BFF) eaten freely together with a fixed amount of lean beef (100 g). In experiment-1, food intake over 30 min and subjective appetite were measured for 120 min. In experiment-2, the same outcomes were measured along with BG, plasma insulin and gut hormones.

**Results:**

The results for boys and girls were pooled as sex was not a factor. In both experiments, children consumed 30–40% less calories at meals with BMP (*P*<0.0001) compared with all other treatments, which were similar. BMP increased satiety, expressed as a change in appetite per kilocalorie, more than all other treatments (*P*<0.0001). FFF resulted in the lowest (*P*<0.0001) glucose and insulin at meal end and post-meal and peptide YY (PYY) post-meal. Blood measures were similar among all other treatments.

**Conclusions:**

The physiological functions of CHO foods consumed *ad libitum* at meal time on food intake, appetite, BG, insulin and gut hormone responses in children is not predicted by the GI.

## Introduction

Over the past 40 years, the consumption of potatoes has decreased by 41%,^1^ which may be a consequence of movements aimed at decreasing serving sizes or the removal of French fries from school cafeterias and other quick service restaurant meals for children.^2, 3^ This may be due, in part, to the increasing evidence in the literature where observational studies show potato consumption may lead to increased risk of obesity.^4^ Moreover, it has led to an increased demand for general dietary advice to replace potatoes with rice and pasta, which may or may not be of lower glycemic index (GI). However, these carbohydrates (CHOs) are rarely eaten alone on a daily basis,^5^ but are commonly consumed in a meal with other foods that lower the GI of the meal when compared with CHO sides eaten alone.^6^ For example, the GI of potatoes was significantly reduced from 93 to 39 when boiled Estima potatoes were served with 62 g of cheddar cheese and from 108 to 54 when mashed potatoes were served with oil, chicken breast and salad in amounts that represent a meal.^5, 6^ Similarly, the GI of rice-based meals is markedly reduced through addition of other meal components such as tofu, eggs and vegetable^7^ or chicken breast, vegetables and oil.^8^ Therefore, these studies lead to the hypothesis that advice based on the GI of fixed amount (50 g) of available CHO, may not be representative of post-prandial satiety and glycemia when CHOs are consumed freely in a mixed meal.^9^ Yet, only one study has reported the effect of providing *ad libitum* access to CHO sources in a meal on energy consumed and postprandial glucose.^10^ Food intake by men given a fixed portion of meat ate 31% and 23% less with free access to mashed potatoes compared with pasta and rice meals, respectively.^10^ No similar study of children has been reported.

The rationale behind the present study was twofold; first, current dietary recommendations regarding CHOs suitable in meals for children are not based on meal studies and second, are heavily dependent on their high GI and their fat content, and not on their overall functionality within a meal. We hypothesized that energy intake, postprandial subjective appetite, blood glucose (BG) and insulin in children following *ad libitum* meals with meat is not predicted from the GI of the CHO. Therefore, the objective of this study was to determine the effects of commonly consumed CHO side dishes such as potatoes, pasta and rice along with a fixed portion of meat on food intake, satiety, BG, insulin and gut hormone response among children (aged 11–13 years) with healthy body weight over a 2-h period.

## Materials and methods

Normal weight children (boys and girls, aged 11–13 years old) born full-term and of normal birth weight participated. This study was performed according to the guidelines in the Declaration of Helsinki. All treatments and procedures were approved by the Human Participants Review Committee, Ethics Review Office, University of Toronto. The recruitment strategies were similar to those reported previously.^11, 12^ An in-person screening was scheduled at the Department of Nutritional Sciences, University of Toronto, where written informed consent was obtained from the parent and child. Height (m) and weight (kg) were measured while in light clothing and without shoes to determine age- and sex-specific body mass index percentiles (between the 15th and 85th percentile), according to the World health Organization growth charts.^13^

### Protocol

Two experiments were conducted. Both experiments followed a within subject, randomized, repeated-measures design. All participants attended five sessions that were scheduled once per week for 5 weeks. The five treatment sessions consisted of *ad libitum* servings of (i) rice, (ii) pasta, (iii) boiled and mashed potato (BMP), (iv) baked French fries (BFF) and (v) fried French fries (FFF) with a fixed amount (100 g) of meatballs.

Study protocol and procedures were similar to those reported previously.^12^ Participants attended the Department of Nutritional Sciences following a 12-h overnight fast, except for water, which was permitted until 1 h before each session. To minimize within subject variability, all participants were scheduled to arrive at the same time and on the same day of the week for each treatment and maintain the same dietary and exercise patterns the evening before each test. On five weekend mornings, each participant arrived at the laboratory between 1100 and 1300 hours, 4 h after which they consumed a standardized breakfast at home consisting of 26 g of Honey Nut Cheerios cereal (General Mills, Inc., Minneapolis, MN, USA), 250 ml of Beatrice 2% milk (Parmalat Canada, Inc., Toronto, ON, Canada) and 250 ml Tropicana orange juice (Tropicana Products, Inc., Bradenton, FL, USA). Participants who did not consume the breakfast at the correct time or reported variances in normal activity and diet the night before were rescheduled. On arrival, before the beginning of each test, participants completed visual analog scales assessing their ‘sleep habits', ‘stress factors', ‘food intake and activity level', physical comfort ‘feeling of fatigue' and ‘motivation to eat' in both experiments. Visual analog scales were administered at baseline (0 min) and at 30, 45, 60, 75, 90, 105 and 120 min in both experiments. In experiment 2, each subject provided a baseline finger-prick capillary blood sample using a Monoejector Lancet device (Sherwood Medical, St Louis, MO, USA) to ensure compliance with fasting instructions. Plasma concentration of glucose was measured with a point-of-care glucose meter (Accu-Chek compact; Roche diagnostics, Laval, QC, Canada). A baseline measurement of >5.5 mmol l^−1^ indicated noncompliance with the fasting instructions, and participants were rescheduled. Following the finger-prick BG measurement, an indwelling intravenous catheter was inserted in the antecubital vein by a registered nurse and a baseline blood sample was obtained. Immediately thereafter, participants were escorted to a feeding room and seated in individual cubicles. Each child was instructed to eat until feeling comfortably full. After the participants were provided with the first plate of 250 g serving of CHO sides with meatballs, additional trays of freshly cooked CHO sides were provided 10 and 20 min later, with removal of the previous tray and were allowed 30 min to complete the meal. The total amount of CHO sides consumed by the children was determined by weighing the amount of food served and left over during the treatment meal by each child.

### Treatment meals

The test meals were prepared in the laboratory kitchen according to package instructions. To avoid starch retrogradation, all treatments were served hot, immediately after removal from the oven or microwave. (i) BMPs were prepared by cooking 250 g of frozen diced potato (McCain Simply Dice, McCain Foods, Florenceville-Bristol, NB, Canada) for 2 min in boiling water, with a potato to water ratio of 1:2.5 and then was transferred to a strainer in order to drain the water, and was mashed with 60 ml of milk (3.25% Beatrice, Parmalat Canada, Inc.) and 15 g unsalted butter (Neilson, Saputo Dairy Products Canada G.P., St-Laurent, QC, Canada). (ii) Pasta was prepared by cooking 225 g of pasta (Kraft Dinner, original, Kraft Canada, Toronto, ON, Canada) for 8 min in boiling water, with a pasta-to-water ratio of 1:2.5. The pasta was subsequently drained and mixed with 80 ml of milk (3.25% Beatrice, Parmalat Canada, Inc.), 15 g unsalted butter (Neilson, Saputo Dairy Products Canada G.P.) and dry cheese mix (provided with package). (iii) Rice was prepared by microwaving 250 g of rice (Uncle Bens white Basmati, Mars, Inc., Houston, TX, USA) for 2 min and then mixed with 10 g unsalted butter (Neilson, Saputo Dairy Products Canada G.P.) and fried rice seasoning. (iv) BFF was prepared by baking 400 g frozen McCain super fries in the oven for 18 min at 450^o^F. (v) FFF was prepared by frying 400 g frozen McCain French fries in the fryer with canola oil (Messina Brands, Markham, ON, Canada) for 5.5 min at 375^o^F. (vi) A fixed portion of lean beef meat was prepared by cooking 100 g meatballs (President's Choice Blue menu lean Italian beef meatballs, Loblaw Companies Limited, Toronto, ON, Canada) in the oven for 12 min at 400^o^F. Treatment composition and characteristics are shown in Table 1.

### Blood parameters

In experiment 2, intravenous blood was collected in lavender capped BD vacutainer tubes (BD Diagnostics, Franklin Lakes, NJ, USA), coated with ethylenediamine tetra acetic acid (EDTA) and immediately treated with dipeptidyl peptidase-4 (DPP-IV), aprotinin, 4-(2-Aminoethyl) benzenesulfonyl fluoride hydrochloride (AEBSF) and other protease inhibitors to prevent proteolytic breakdown of glucagon-like peptide-1 (GLP-1), peptide YY (PYY) and active ghrelin hormones at baseline (0 min) and at 30, 60 and 120 min after the meals. The tubes were centrifuged at 2000 RCF (Micro high-speed refrigerated centrifuge, VS-15000CFNll, Vision Scientific Co., Ltd., Daejeon, South Korea) for 15 min at 4 °C. Plasma samples were aliquoted to Eppendorf tubes and stored at −80 °C for analysis. Plasma concentrations of active GLP1 (intra-CV: <8% inter-CV: <5% CAT#EGLP-35K), acylated ghrelin (intra-CV: <2% inter-CV: <8% CAT#EZGRT-89K) and PYY (intra-CV: <6% inter-CV: <8% CAT#EZHPYYT66K) were measured with ELISA kits (Millipore, Billerica, MA, USA). Plasma insulin concentration (intra-CV: <3% inter-CV: <6% 80-INSHU-E01.1, E10.1) was measured using ELISA kits (Alpco, Salem, NH, USA).

### Statistical analysis

Analyses were conducted using SAS 9.3 (SAS Institute Inc., Cary, NC, USA). Food Intake was analyzed using one-factor repeated-measures analysis of variance (ANOVA) with a Tukey–Kramer's *post hoc* analysis test. Effects of time, treatment and treatment × time interaction on subjective appetite, BG and gut hormones were analyzed using two-way repeated-measures ANOVA, followed by Tukey's *post hoc* test. When an interaction was statistically significant, one-way ANOVA was conducted followed by Tukey's *post hoc* test to investigate the effect of treatment differences at each time point. The effect of treatment on pre-meal (0 min), meal end (30 min) and post-meal (30–120 min) BG, insulin, GLP1, PYY and active ghrelin was tested via one-way repeated-measures ANOVA followed by Tukey's *post hoc* test. Pearson's correlation coefficients were used to detect associations between dependent measures. Average appetite was calculated as previously reported.^14^ All results are presented as mean±standard error of the mean (s.e.m.). Statistical significance was concluded with a *P*-value less than 0.05.

## Results

### Participants

Subject characteristics are presented in Table 2. For experiment 1, 14 boys and 9 girls were recruited; however, 2 boys and 1 girl did not complete all sessions due to time constraints. The final number of boys and girls included in the analysis was 12 and 8, respectively. For experiment 2, 7 boys and 6 girls were recruited; however, 1 boy withdrew from the study due to holiday plans. Therefore, 6 boys and 6 girls were included in the final analysis of experiment 2. The reported results were pooled for both boys and girls as sex was not a factor in experiment 1 (*P*=0.4227) or experiment 2 (*P*=0.3615).

### Food Intake

The consumption of the five CHOs at meals by weight (g) and calories (kcal) eaten are shown in Table 3. The weight (g) of BFF and FFF consumed was significantly lower (*P*<0.0001) than pasta in both experiments 1 and 2 (Table 3). However, owing to the different energy densities of the five CHO sources energy intake (kcal), energy intake was lower (*P*<0.0001) after the BMP meal compared with rice, pasta, BFF and FFF meals, which were similar in both experiments 1 and 2 (Table 3). Because the addition of meat was through a fixed amount, the total cumulative weight and energy intake followed the same pattern as the side dishes alone (Table 3). Water intake was not different (*P*=0.961) among the meals (data not shown). Average palatability [(pleasantness+taste+texture)/3] ratings were similar among CHO meals (*P*=0.1924).

### Subjective appetite

In experiment 1, average appetite (0–120 min) was significantly affected by time (*P*<0.0001), but not treatment (*P*=0.6212) or treatment-by-time interaction (*P*=0.4506). Pre-meal appetite was highest at baseline, averaging 81.3 mm and decreased after the treatment meal to 9.2 mm at 30 min and slowly rose to a mean of 31.6 mm at the end of the study period (120 min). There were no differences between any of the treatments at any time post-meal. In experiment 2, the response in average appetite (0–120 min) was reproduced as in experiment 1.

**Figure 1:**
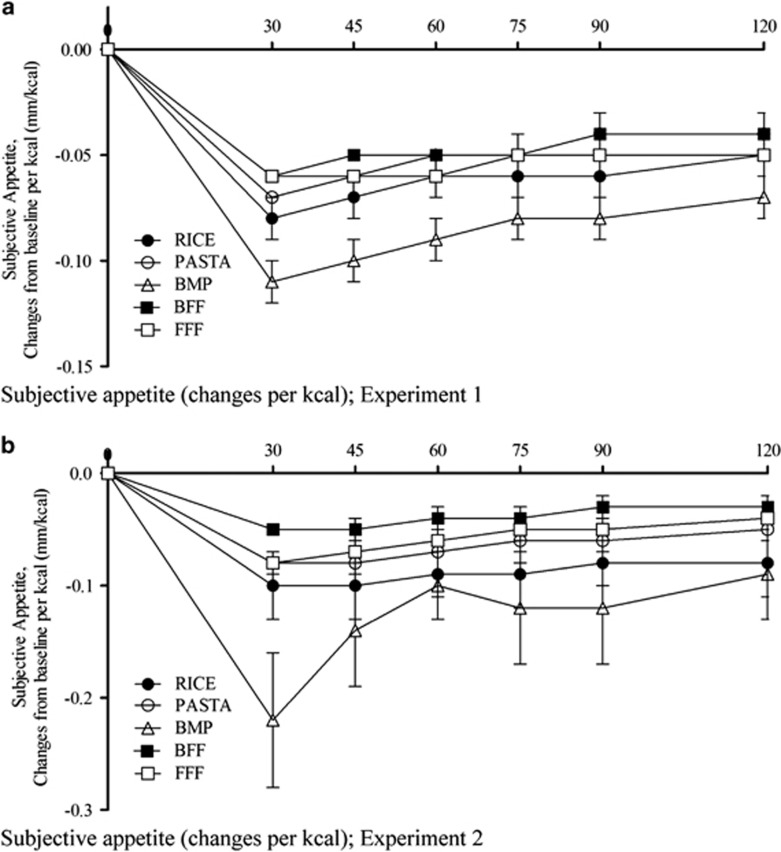
Effect of treatments on average subjective appetite (changes from baseline per kcal) over time. (**a**) Experiment 1, (**b**) experiment-2. Values are means with their standard errors represented by vertical bars (*n*=20: experiment 1, *n*=12: experiment 2). All measures changed over time (*P*<0.0001), and subjective appetite was affected by treatment in both experiments 1 and 2 (*P*<0.05, by two-way ANOVA; BMP resulted in lower subjective appetite (changes from baseline per kcal compared to all other treatments) in experiment 1 (*P<*0.001) and experiment 2 (*P*<0.001)).

Expressed as a change in appetite per kilocalorie of the treatment, the average appetite scores (0–120 min) were affected by time (*P<*0.001) and treatment (*P<*0.001), but there was no time and treatment interaction (*P*=0.1985) in either experiment 1 or 2. All treatments reduced post-meal average appetite, but post-meal appetite per kcal was lowest following consumption of the BMP (*P<*0.001) compared with rice, pasta, BFF and FFF in experiments 1 and 2 (Figure 1).

### Blood glucose (experiment 2)

**Figure 2:**
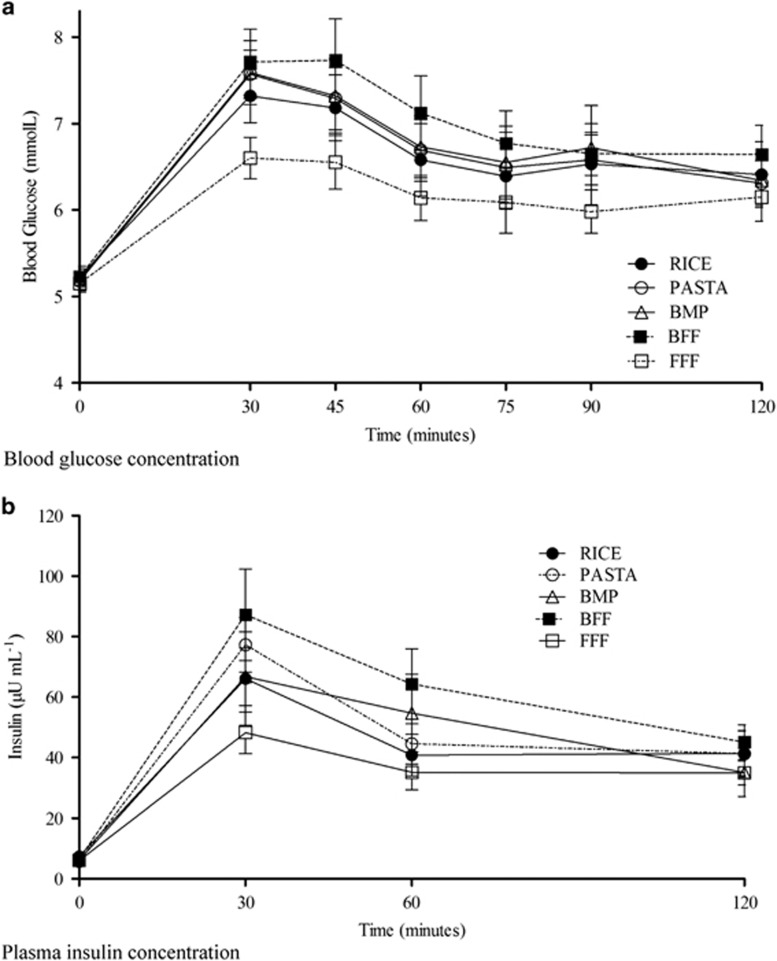

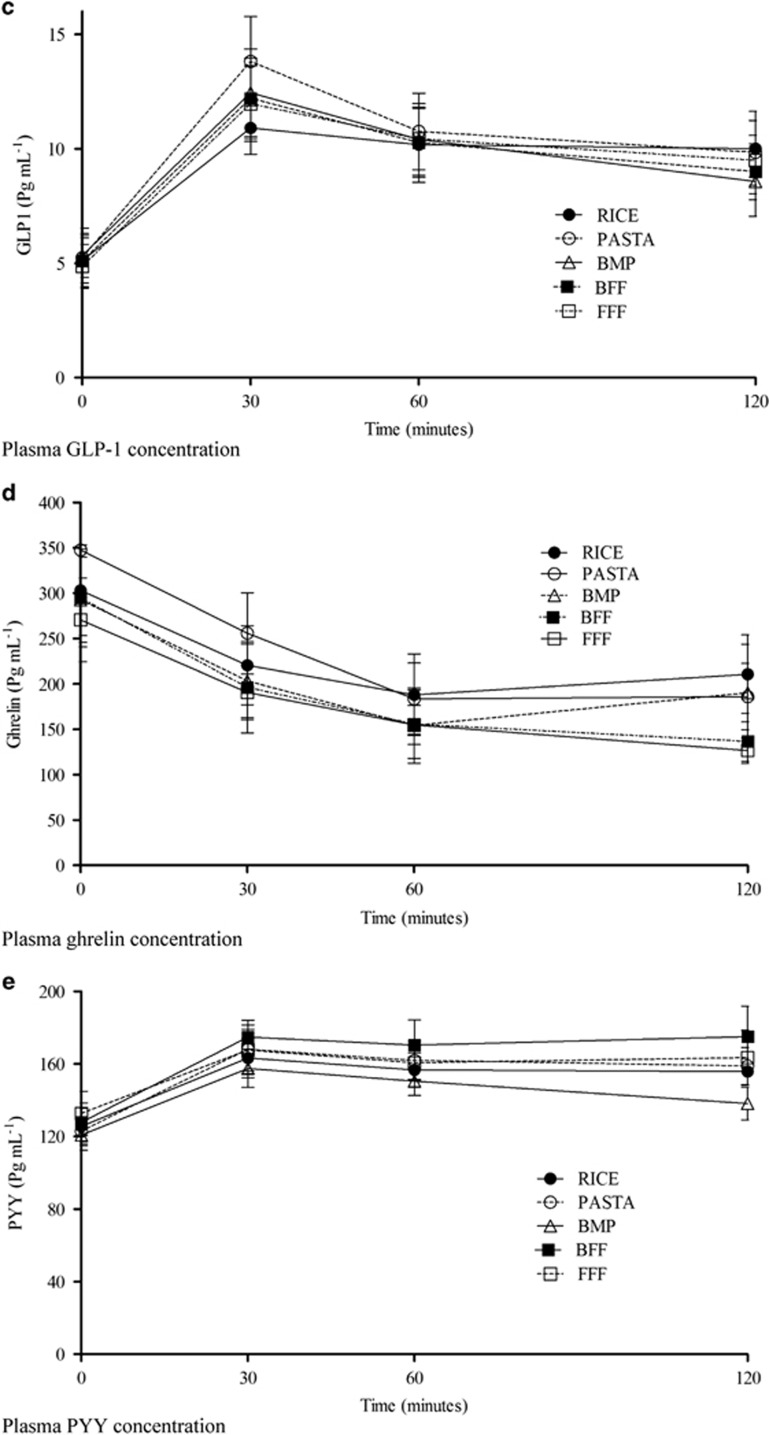
Effect of the teatments on (**a**) blood glucose, (**b**) Insulin, (**c**) ghrelin, (**d**) GLP1 and (**e**) PYY concentration over time (Experiment 2). Values are means (*n*=12), with their standard errors represented by vertical bars. All measures changed over time (*P*<0.0001), but only blood glucose, insulin, ghrelin and PYY were affected by treatment (*P*<0.05; by two-way ANOVA).

BG concentrations (0–120 min) were affected by time (*P<*0.0001) and treatment (*P<*0.0001), but there was no time and treatment interaction (*P*=0.9611; Figure 2a). The BG response peaked at 30 min after treatment meals, but there was a difference in the pattern of increase among the treatments over 120 min (Figure 2a). At 30 min, FFF resulted in a lower peak BG concentration compared with BFF, BMP and pasta (*P*=0.0039*,* Table 4). During the entire post-meal period (30–120 min), FFF sustained lower *(P*<0.0001) BG concentrations compared with all other treatments (Table 4). Basal concentrations of glucose before meal ingestion (0 min) were not different between the treatments (*P*=0.1861; Table 4).

### Plasma insulin concentrations (Experiment 2)

Insulin concentrations (0–120 min) were affected by time (*P*<0.0001) and treatment (*P*=0.0178), but there was no time and treatment interaction (*P*=0.2988). The insulin concentrations were highest at 30 min after the treatment meals (Figure 2b). At 30 min, FFF resulted in lower peak insulin concentration compared with BFF (*P*=0.0018; Table 4), and mean insulin concentrations during the entire post-meal (30–120 min) period was lower after FFF compared with BFF (*P*=0.0018; Table 4). Baseline concentrations of insulin before meal ingestion (0 min) were not significantly different between the groups (*P*=0.2988; Table 4).

### Plasma GLP1 concentrations (Experiment 2)

Plasma GLP1 concentrations (0–120 min) were affected by time (*P*<0.0001), but not by either treatment *(P*=0.4718) or time and treatment interaction (*P*=0.8034; Figure 2c). GLP1 concentration was lowest at baseline and increased after the treatment meals to 30 min and remained at similar levels for the duration of the study period. The post-meal (30–120 min) average GLP1 concentrations were found to be similar *(P*=0.4285) between treatment meals (Table 4).

### Plasma ghrelin concentrations (Experiment 2)

Plasma ghrelin concentrations (0–120 min) were affected by time (*P<*0.0001) and treatment (*P*=0.0265), but not by time and treatment interaction (*P*=0.9318). Baseline concentrations of ghrelin before meal ingestion were not different between the treatments (*P*=0.1951; Table 4). Pre-meal ghrelin concentration was highest at baseline and decreased after all CHO treatments to a low at the end of the meal at 30 min and gradually rose until 120 min (Figure 2d). During the entire post-meal (30–120 min) period, mean ghrelin concentrations were reduced most after FFF compared with pasta (*P*=0.0168; Table 4).

### Plasma PYY concentrations (Experiment 2)

Plasma PYY concentrations (0–120 min) were affected by time (*P*<0.0001) and treatment (*P*=0.0089), but not by time and treatment interaction (*P*=0.9785). Pre-meal PYY increased after the treatment meals to 30 min and remained at similar levels for the duration of the study period (Figure 2e). Baseline concentrations of PYY before meal ingestion were not different between the treatments (*P*=0.2441; Table 4). During the entire post-meal (30–120 min) period, mean plasma PYY concentrations were lower for BMP (*P*=0.0113) compared with FFF and BFF meal (Table 4).

### Relations between dependent measures

The amount of CHO intake was positively associated with post-meal blood glucose incremental AUC (*r*=0.39, *P* <0.0001), and insulin iAUC (*r*=0.06, *P*=0.0211). Total calorie intake was not associated with post-meals average appetite (*r*=−0.06, *P*=0.4117). Palatability ratings of CHO meals was not associated with food intake (*r*=0.12, *P*=0.9874). Blood glucose AUC was positively associated with insulin AUC (*r*=0.3, *P*=0.0237), but not with GLP1 and PYY.

## Discussion

The results of this study support the hypothesis that energy intake and glycemic response during and following an *ad libitum* meal containing potatoes, rice or pasta is not predicted based on their GI. Boiled mashed potato co-ingested with meat resulted in ~40% lower energy (kcal) intake, with similar postprandial glucose, insulin, GLP1 and ghrelin compared with BFF, pasta and rice. FFF resulted in lower post-meal glucose compared with all other treatments and lower insulin than BFF, but similar food intake and post-meal satiety to pasta, rice and BFF.

The boiled mashed potato meal reduced food intake at the meal and increased post-meal satiety, compared with all other treatments (*P<*0.001), when expressed as a change in appetite per kcal of the CHO meal. This lower food intake was consistent with the results of a study in adult men showing that the energy consumed at an *ad libitum* potato meal with 150 g of meat was 31% and 23% lower compared with pasta and rice meals, respectively.^10^ Although GI and appetite scores have been reported to be inversely associated over 2 h following treatments,^15^,^16^ the blood glucose was not higher at meal termination after BMP compared with rice but may reflect the role of combining protein and CHO in termination via hypothalamic mechanisms regulating food intake.^9, 17, 18^

Although food intake was markedly lower in the BMP treatment group in comparison to other CHO sides, post-meal glucose and insulin were not lower at meal end (30 min) and post-meal (30–120 min). This is in contrast to the study conducted on men,^10^ which showed that food intake at a boiled potato meal with meat was 40% lower compared with rice and pasta with meat. Furthermore, postprandial insulin was markedly reduced and glucose tended to be lower after the boiled potato meal, reflecting their reduced CHO intake.^10^ It is difficult to explain why post-prandial glucose and insulin were not lower in the present study as the children also consumed 30% less CHO. The only difference in the potato treatment was that fresh boiled potato was consumed by the men and the children consumed boiled frozen and mashed potatoes. Therefore, it remains to be determined if children would respond similarly to eating freshly boiled potatoes or whether adults respond similar to children after consuming BMP, FFF or BFF with meat. The lower energy intake after boiled potatoes in meals consumed by the men was not made up at a later meal 4 h later,^10^ but the effects of mashed potatoes at a meal on later meal intakes in children remain to be determined.

Postprandial glucose and insulin concentrations are primarily determined by dietary CHO.^6^ Therefore, it is not surprising that the FFF meal resulted in the lowest post-meal blood glucose as they are highest in fat and lowest in CHO (Table 1). FFF provided a lower CHO load at the meals compared with all other treatments (FFF=117 g, BFF=199 g, rice=161 g and pasta=162 g), as some leaching of the starch constituents (namely amylose) into the frying oil occurs during frying.^19^ Furthermore, the consumption of fat with CHO foods delays starch degradation by slowing gastric emptying^20^ and reduces postprandial glucose with no effect on the insulin response.^7^ Pre-frying and freezing increases the amount of resistant starch in FFF, while amylose may also react with lipids to form amylose-lipid complexes^21, 22^ that are slowly digested.^23, 24^ In comparison to FFF, BFF resulted in higher blood glucose and insulin responses but were expected because of their higher CHO content. Baking uses dry heat,^25^ and causes loss of water, which in turn results in concentrating free sugars.^24^ Moreover, starch gelatinization through cooking and processing increases the susceptibility of starch to digestive enzymes, making the BFF more readily digested by amylase enzymes in comparison to starch in its native form.^26^

In addition to insulin and glucose, GLP1, ghrelin and PYY were measured to gain insight on the mechanism of food intake responses. The most favorable metabolic profile was found after the FFF meal, as reflected by a larger suppression of ghrelin, an orexigenic appetite-stimulating hormone,^27^ and increase in PYY, an anorexogenic hormone,^27^ combined with a sustained and lower insulin and postprandial blood glucose.^28^ FFF suppressed ghrelin, more compared with pasta and increased PYY more than after BMP. Although food intake was much lower after BMP, the decrease in ghrelin was similar to other treatments, resulting from a stronger satiety effect of the calories consumed. GLP1 has a role in regulation of blood glucose and food intake, but did not differ by treatment (Figure 2c and Table 4).

The role of energy density and volume as determinants of food intake was not consistent with the volumetric hypothesis of meal intake regulation. Several studies have shown that adding water within a meal eaten freely reduces energy intake, but not amount of food consumed.^29, 30^ Consistent with this hypothesis, the highest weight and lowest energy intake was at the BMP meal (598 g vs 507kcal in experiment 1 and 545 g vs 517kcal in experiment 2). However, the energy density of pasta was also comparatively low, but the children compensated by eating more weight to achieve similar energy intakes as at meals with other CHOs. Palatability of food is another major determinant of children's energy intake.^31^ However, palatability scores for the potato, rice and pasta meals were found to be similar in this study (data not shown), and food intake was not influenced by palatability of the meals (*r*=0.087, *P*=0.133).

In summary, the results of this study suggest that applying the GI of CHOs to predict their postprandial glycemic effects in a mixed meal can be misleading and may lead to counterproductive dietary guidance. Indeed, the results, in contrast to recommendations for children to avoid eating potatoes, support their consumption instead of other commonly consumed CHOs such as rice and pasta. Furthermore, removing potatoes from meals is of concern, because substituting other CHOs reduces meal nutrient intake as potatoes contain a balanced high nutrient-to-energy ratio.^32^ Potatoes provide valuable shortfall nutrients for children as well as adults including potassium, magnesium, vitamin C, vitamin B6, iron and fiber and bioactive phenolics. Potato is also a source of high-quality protein and has favorable ratios of high nutrient-to-energy density^9^ and protein calories-to-total calories.^33^

In conclusion, the physiological functions of CHOs consumed *ad libitum* at meal-time on food intake, satiety, glycemic, insulin and gut hormone responses in children cannot be judged by their GI alone.

## Figures and Tables

**Table 1:** Nutritional composition of potatoes, rice, pasta and meat

*per 100 g*	*Rice*	*Pasta*	*BMP*	*BFF*	*FFF*	*Meatballs*
Protein (g)	3.86	5.45	1.29	3.82	3.83	17.60
Fat (g)	4.48	2.54	3.93	6.11	13.20	9.40
Carbohydrate (g)	36.00	25.60	13.50	43.90	30.60	8.20
Calories (kcal)	200.00	147.00	95.00	246.00	256.00	188.00
Volume^10^	112.00	104.00	90.00	108.00	124.00	95.00
Glycemic index (WB)	57	64	91	64	70	N/A
Moisture (g)	55.10	65.30	80.50	44.50	51.20	49.40

Abbreviations: BFF, baked French fries; BMP, boiled and mashed potato; FFF, fried French fries; N/A, not applicable; WB, white bread.

Values presented per 100 g.

**Table 2:** Baseline characteristics of participants

	*Experiment 1*			*Experiment 2*		
	*Boys (*n=*12)*	*Girls (*n=*8)*	P	*Boys (*n=*6)*	*Girls (*n=*6)*	P
Age (years)	12.08±0.23	11.88±0.35	0.6084	12.3±0.33	11.7±0.33	0.1877
Weight (Kg)	45.65±1.43	48.10±2.22	0.3430	46.9±2.14	45.3±2.05	0.5794
Height (cm)	157.13±2.32	159.56±2.47	0.4935	157.4±2.94	154.3±1.98	0.4048
BMI (kg m^−2^)	18.46±0.40	18.83±0.41	0.5437	18.9±0.45	18.9±0.69	0.9369
BMI-for-age percentile	55.71±6.94	57.73±7.56	0.8501	59.6±8.39	61.7±7.7	0.8577

Abbreviations: ANOVA, analysis of variance; BMI, body mass index.

Data are presented as means±s.e. One-factor ANOVA was used to compare group (boys vs girls) baseline characteristics.

**Table 3:** Treatment effects on food intake (weight and kcal)

*Treatments*	*Experiment 1*			*Experiment 2*		
	*CHO amount (g)*	*Meat amount (g)*	*Cumulative amount (g)*	*CHO amount (g)*	*Meat amount (g)*	*Cumulative amount (g)*
Rice	424.4±77.9 bc	98.6±0.8	523.1±78.3 bc	423.6±33.4 a	103.6±1.4	527.2±33.5 a
Pasta	598.8±77.5 a	99.9±1.1	698.8±77.9 a	607.4±41.5 b	103.8±1.2	711.2±41.8 b
BMP	534.6±104.3 ab	102.7±1.1	637.2±104.1 ab	545.2±51.9 b	101.1±1.0	646.3±52.1 b
BFF	435.2±40.4 bc	99.6±1.3	535.2±40.6 bc	415.4±28.4 a	101.2±1.3	516.6±28.1 a
FFF	356.3±36.1 c	100.1±1.7	456.4±36.8 c	367.5±24.5 a	103.8±1.2	471.3±24.4 a
	*P*<0.0001	*P*=0.2456	*P*<0.0001	*P*<0.0001	*P*=0.2822	*P*<0.0001
	*CHO calories*	*Meat calories*	*Cumulative calories*	*CHO calories*	*Meat calories*	*Cumulative calories*
Rice	848.8±155.8 a	185.3±1.5	1034.2±156.6 a	847.1±66.7 b	194.8±2.5	1041.9±67.1 a
Pasta	880.3±113.9 a	187.9±1.9	1068.3±114.7 a	892.9±61.1 ab	195.2±2.2	1088.1±60.4 a
BMP	507.9±99.1 b	193.1±1.9	700.8±98.6 b	517.9±49.3 c	190.1±1.9	707.9±49.6 b
BFF	1070.7±99.4 a	187.9±2.5	1258.6±99.5 a	1021.8±69.9 a	190.3±2.5	1212.1±69.4 a
FFF	912.2±92.4 a	188.2±3.2	1100.4±92.7 a	940.8±62.6 ab	195.2±2.3	1136.1±62.5 a
	*P*<0.0001	*P*=0.2836	*P*<0.0001	*P*<0.0001	*P*=0.2836	*P*<0.0001

Abbreviations: ANOVA, analysis of variance; BFF, baked French fries; BMP, boiled and mashed potato; CHO, carbohydrate; FFF, fried French fries.

Data are presented as means+s.e. (*n*=20 in experiment 1; *n*=12 in experiment 2). Values in the same column with different superscript letters are significantly different (one-way ANOVA followed by Tukey's *post hoc* test; *P*<0.05). Cumulative intake=amount of CHO (grams or calories) + amount of meat (grams or calories) consumed. As presented in the captions, letters a, b and c indicate significance level of *P*<0.05 between the groups (by one-way ANOVA followed by Tukey's *post hoc* test). *P*-values presented in the table are the overall *P*-values for one-way ANOVA.

**Table 4:** Effect of carbohydrate treatments on blood glucose, insulin, PYY, Ghrelin and GLP1 concentrations at baseline (0 min), after meal (30 min) and post-meal (30–120 min): experiment-2

*Treatments*	*Experiment 2*		
	*Before meal (0 min)*	*End meal (30 min)*	*Post-meal (30–120 min)*
*Blood glucose (mmol l^−1^)*			
Rice	5.22±0.1	7.31±0.3 ab	6.73±0.2 a
Pasta	5.18±0.1	7.56±0.3 a	6.82±0.1 a
BMP	5.21±0.1	7.59±0.4 a	6.87±0.2 a
BFF	5.23±0.1	7.70±0.4 a	7.09±0.2 a
FFF	5.15±0.1	6.60±0.2 b	6.25±0.1 b
	*P*=0.1861	*P*=0.0039	*P*<0.0001
			
*Plasma insulin (μU ml^-1^)*			
Rice	7.37±1.1	66.09±15.4 ab	49.44±6.3 ab
Pasta	5.79±0.6	77.29±9.1 ab	54.39±5.2 ab
BMP	6.14±0.7	66.76±9.6 ab	52.18±6.2 ab
BFF	6.41±0.8	87.21±15.1 a	65.53±7.1 a
FFF	6.01±0.8	48.20±6.9 b	39.43±3.3 b
	*P*=0.2988	*P*=0.0018	*P*=0.0018
			
*GLP1 (pg ml*^−*1*^)			
Rice	5.12±0.9	10.91±1.2	10.36±0.8
Pasta	5.24±1.3	13.82±1.9	11.47±1.1
BMP	5.34±0.9	12.85±1.9	10.47±0.1
BFF	5.10±1.1	12.21±1.8	10.49±0.9
FFF	4.87±0.9	11.97±1.7	10.63±0.8
	*P*=0.2988	*P*=0.3955	*P*=0.4285
			
*Active ghrelin (pg ml^-1^)*			
Rice	303.2±49.9	220.6±43.4	206.49±24.7 ab
Pasta	347.7±52.3	255.8±44.7	208.55±23.8 a
BMP	292.6±46.9	203.5±40.6	182.73±25.7 ab
BFF	294.7±54.1	196.4±50.2	162.99±21.9 ab
FFF	270.6±46.1	190.7±29.8	157.47±13.5 b
	*P*=0.1951	*P*=0.1514	*P*=0.0168
			
*PYY (pg ml^-1^)*			
Rice	125.6±9.8	163.3±10.8	158.61±5.2 ab
Pasta	122.9±7.9	168.1±13.3	162.98±6.4 ab
BMP	120.8±8.3	157.5±10.4	148.72±5.3 a
BFF	127.8±10.6	174.8±9.3	173.41±7.7 b
FFF	132.6±12.2	167.6±11.4	168.87±6.8 b
	*P*=0.2441	*P*=0.2054	*P*=0.0113

Abbreviations: ANOVA, analysis of variance; BFF, baked French fries; BMP, boiled and mashed potato; FFF, fried French fries.

Effect of the treatments on mean glucose, insulin, GLP1, ghrelin and PYY concentrations at baseline/before meal (0 min), after meal (30 min) and post-meal (30–120 min). Values are means (*n*=12), with their standard errors. Mean values with unlike letters were significantly different (*P*<0.05; one-way ANOVA followed by Tukey–Kramer *post hoc* test).

## Notes

GHA has served as a consultant to McCain Foods Canada. The remaining authors declare no conflict of interest.
